# Apremilast ameliorates methotrexate-induced renal injury in rats: role of TLR4/NF-κB/P38 MAPK/caspase-3 and Nrf2/HO-1 signaling pathways

**DOI:** 10.1007/s00210-025-04846-w

**Published:** 2025-12-15

**Authors:** Reham H. Mohyeldin, Ehab E. Sharata, Mahmoud Abdelnaser, Mina Ezzat Attya, Al Shaimaa Mahmoud Kotb, Remon Roshdy Rofaeil

**Affiliations:** 1 Department of Pharmacology & Toxicology, Faculty of Pharmacy, Deraya University, Minia, 61111 Egypt; 2 Department of Biochemistry, Faculty of Pharmacy, Deraya University, Minia, 61111 Egypt; 3 Department of Pathology, Faculty of Medicine, Minia University, Minia, 61519 Egypt; 4 Department of Pathology, Faculty of Medicine, Minia National University, Minia, 61519 Egypt; 5 Medical Physiology Department, Faculty of Medicine, Minia University, Minia, 61519 Egypt; 6 Department of Medical Pharmacology, Faculty of Medicine, Minia University, Minia, 61511 Egypt

**Keywords:** MTX, Apremilast, Kidney injury, Caspase-3, TLR4, Nrf2

## Abstract

This study aimed to assess the preventive potential of apremilast (APRE) against methotrexate (MTX)-induced renal damage in rats through modulation of nuclear factor erythroid 2-related factor 2/heme oxygenase-1 (Nrf2/HO-1) signaling and toll-like receptor 4/nuclear factor-kappa B/p38 mitogen-activated protein kinase/caspase-3 (TLR4/NF-κB/p38 MAPK/Caspase-3) signaling pathways. Four groups of male Wistar albino rats were assigned: control, APRE, MTX, MTX + APRE. Histopathological investigation and biochemical analysis of the serum renal damage indicators (urea and creatinine) were used to evaluate the renal toxicity of MTX. Testing for renal malondialdehyde (MDA) and reduced glutathione (GSH) was conducted. The levels of renal tumor necrosis factor-alpha (TNF-α), interleukin-6 (IL-6), Nrf2, HO-1, and cleaved caspase-3 were measured using the ELISA method. Using an immunohistochemistry method, the expression of NF-κB p65 in the kidney was investigated. Western blotting was used to examine the expression of TLR4 and p38 MAPK proteins. MTX administration resulted in significant renal injury, as evidenced by elevated serum urea and creatinine levels. The kidneys were significantly affected as evidenced by histopathological alterations and increased levels of renal MDA, TNF-α, IL-6, Bcl-2-associated x (Bax), and cleaved caspase-3, alongside decreased levels of GSH and B-cell lymphoma 2 (Bcl-2) expression. These outcomes were linked to inhibition of Nrf2/HO-1 signaling and activation of the TLR4/NF-κB/p38 MAPK/Caspase-3 pathway. Co-treatment with APRE at 20 mg/kg/day for 21 days markedly improved all biochemical and pathological alterations evoked by MTX, demonstrating significant nephroprotective effects. Apremilast inhibits methotrexate’s harmful effects on the kidneys by activating signaling cascades that include Nrf2/HO-1, while simultaneously downregulating TLR4/NF-κB/p38 MAPK/Caspase-3.

**Supplementary Information:**

The online version contains supplementary material available at 10.1007/s00210-025-04846-w.

## Introduction

Psoriasis affects more than 125 million people worldwide and is characterized by persistent inflammation (Takeshita et al. 2017). The skin symptoms of psoriasis can be accompanied by systemic complications such as psoriatic arthritis, heart disease, and metabolic syndrome. Multiple factors, including heredity, environmental exposure, and immune system dysregulation, contribute to the disease’s etiology. When the condition is moderate to severe, systemic treatments are usually necessary to slow its course and enhance patients’ quality of life (Raharja et al. 2021). Systemic treatments, such as cyclosporine, methotrexate (MTX), and biological medicines, are used to treat mild to severe illnesses. The immunomodulatory function and relatively low cost of MTX keep it at the forefront of systemic agent therapy. Over 70% of patients with psoriatic arthritis and psoriasis continue to use MTX, making it the most often used disease-modifying and rheumatic medication (Menter et al. 2019; Fiore et al. 2018). Although MTX is effective in treating psoriasis, it has a risk of causing multiorgan toxicity, especially in the kidneys and liver, which limits its use for long-term or high-dose use (Howard et al. 2016). Prior research has shown that nephrotoxicity frequently originates from reduced renal tubular function and glomerular damage; elevated blood creatinine, uremia, and hematuria may occur after high-dose MTX therapy (Sparks et al. 2021). It is thought that these negative consequences are caused by a series of events that weaken the structure and function of the kidneys, including oxidative stress, mitochondrial malfunction, inflammation, and apoptosis (Rofaeil et al. 2023; Erboga et al. 2015).

According to earlier studies, a soluble enzymatic mechanism in the liver converts MTX to its main extracellular metabolite, 7-hydroxymethotrexate. Polyglutamated MTX is kept inside cells. Low folate levels and buildup of MTX polyglutamates can occur with either long-term medication therapy or large doses of medicine. Low folate levels and buildup of MTX polyglutamates can occur with either long-term medication therapy (weekly doses of 7.5–25 mg for chronic conditions) (Hawwa et al. 2015), or large doses of medicine (high-dose MTX therapy, typically defined as ≥ 500 mg/m^2^, commonly used in cancer treatment) (Howard et al. 2016). Moreover, high-dose MTX (≥ 1 g/m^2^) is particularly associated with significant polyglutamate accumulation and increased risk of nephrotoxicity, with serum creatinine elevations occurring in 1.8–12% of patients receiving such regimens (Widemann et al. 2004).

Evidence suggests that MTX may reduce cellular NADPH availability by inhibiting cytosolic nicotinamide adenosine diphosphate (NAD[P])-dependent dehydrogenases and the NADP malic enzyme (Vogel et al. 1963). Glutathione reductase normally uses NADPH to keep cytoplasmic glutathione in its reduced form; this cytosolic antioxidant protects cells from reactive oxygen species (ROS). Because MTX promotes a dramatic drop in glutathione (GSH) levels, the antioxidant enzyme defense mechanism becomes less efficient, making cells more vulnerable to reactive oxygen species (ROS) (Caetano, et al. 1997). In addition to being direct cytotoxic agents, these ROS also activate important inflammatory signaling cascades of the TLR4/NF-κB/p38 MAPK/Caspase-3 axis upstream (Younis et al. 2021; Abdelnaser et al. 2025a). Toll-like receptor 4 (TLR4) is upregulated in response to ROS because it is especially vulnerable to oxidative damage and cellular debris. After TLR4 is activated, nuclear factor-kappa B (NF-κB) starts intracellular signaling cascades that cause it to go to the nucleus and produce pro-inflammatory cytokines, including TNF-α, IL-6, and IL-1β (Ali et al. 2017; Hassanein et al. 2018). Parallel p38 MAPK activation increases the transcription of apoptotic genes and intensifies the inflammatory response. The death of renal tubular cells results from the activation of caspase-3, a crucial apoptotic executor, caused by this pro-inflammatory milieu. This oxidative-inflammatory-apoptotic loop’s prolonged activation causes chronic injury to the kidneys in addition to harming tissue structure and function. Therefore, the TLR4/NF-κB/p38 MAPK/Caspase-3 pathway is crucially triggered upstream by MTX-induced ROS, connecting oxidative stress to inflammation and apoptosis in renal damage (Aladaileh et al. 2019). Taken together, these facts demonstrate the critical need for further treatments that might lessen the risks to organs caused by MTX without sacrificing its efficacy in treating psoriasis.

Apremilast (APRE) is an orally selective phosphodiesterase-4 (PDE4) inhibitor that provides a new way to treat moderate to severe plaque psoriasis and psoriatic arthritis; it was authorized by the FDA in 2014 (Al-Harbi et al. 2023). APRE’s ability to modulate inflammatory and oxidative stress pathways has made it a popular candidate for a protective role against many forms of drug-induced toxicity (Liang et al. 2021). In research examining acute lung damage caused by lipopolysaccharide in rats, APRE therapy markedly reduced lung inflammation. The Nrf2/HO-1 signaling pathway was activated, which resulted in decreased oxidative stress and the inhibition of pro-inflammatory cytokines, including TNF-α and IL-6 (Al-Harbi et al. 2023; Khallaf et al. 2025). Moreover, APRE has demonstrated the capacity to mitigate doxorubicin-evoked cardiac injury by reducing oxidative stress and inflammation through suppression of the NF-κB pathway, leading to reduced synthesis of inflammatory mediators and safeguarding against cellular damage (Imam et al. 2018a; Mohyeldin 2025). Comprehending the interplay among these pathways gives options for targeted therapeutic intervention with drugs such as APRE and offers important insight into the molecular basis of MTX toxicity. It is also noteworthy that APRE may be able to lessen the harm that MTX causes to organs because of its pharmacologically intelligent complementing therapeutic properties in psoriasis treatment. Therefore, this study aimed to evaluate, for the first time, the potential of APRE to mitigate MTX-induced renal damage by modulating the interplay between oxidative, inflammatory, and apoptotic signaling pathways, particularly involving TLR4/NF-κB/p38 MAPK/Caspase-3 and Nrf2/HO-1 pathways, as it is expected to offer translational insights into the combinatory use of MTX and APRE in clinical settings, minimizing adverse effects while maximizing therapeutic outcomes in autoimmune diseases.

## Materials and methods

### Drugs and chemical agents

The supplier of methotrexate (MTX) was Mina Pharm Pharmaceuticals in Cairo, Egypt. The supplier of Apremilast (APRE) was Sigma-Aldrich Chemical Co., located in Missouri, USA. Every other chemical reagent used in this work was of the best grade that could be purchased for use.

### Animals

The male Wistar albino rats were 7–9 weeks old and weighed 180–200 g. We sourced our rats from Egypt’s National Research Centre in Giza. To help the rats adjust to their new environment, we gave them free access to water and food pellets for a week before the experiment. In a controlled setting, the rats were kept under a 12-h light/dark cycle, with a temperature of 25 ± 2 °C and a humidity level of 45 ± 5% (Mohyeldin et al. 2025). Compliance with the ARRIVE (Animal Research: Reporting of In Vivo Experiments) standards was maintained during all animal experiments included in this study. Further, the animal protocols in this work adhered to the NIH Guide (8th edition, National Research Council, 2010) for the Care and Use of Laboratory Animals and were approved by the Institutional Ethical Committee of Deraya Center for Scientific Research, Deraya University, Egypt (permission number: DCSR-04025–51).

### Methodology of treatment

**Fig. 1:**
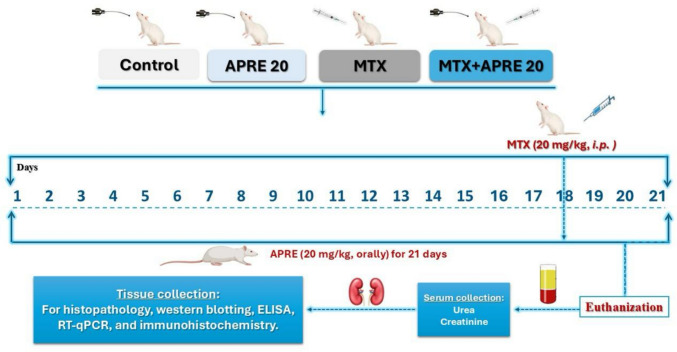
The representative graphical abstract of the treatment protocol

Four groups of 10 rats were randomly selected from the animals. Each group underwent the following treatment protocols, as shown in Fig. 1:

- Control: The rats were given a daily oral dose of normal saline for 21 days.
- APRE 20: Rats were given APRE orally at a dose of 20 mg/kg/day for 21 days.
- MTX: A single intraperitoneal injection of MTX at a dosage of 20 mg/kg was administered to rats on the 18th day of the study period (Ali et al. 2017).
- MTX + APRE: Rats were treated with APRE orally at 20 mg/kg/day for 21 days, along with a single intraperitoneal dose of MTX (20 mg/kg) administered on the 18th day (Imam et al. 2018a; Imam et al. 2019).

All rats received the drugs dissolved in normal saline at a volume of 2 mL/200 g body weight either orally or I.P.

A 21-day course of APRE at a dosage of 20 mg/kg/day was chosen based on recent studies that showed that APRE reduced the risk of cardiac toxicity caused by doxorubicin and lung damage caused by carfilzomib (Imam et al. 2018a; Imam et al. 2019). Several investigations found that the dosage of MTX that was used caused harm to the kidneys (Ali et al. 2017).

### Specimen collection and preparation

An intraperitoneal injection of urethane (1.3 g/kg) dissolved in distilled water was used to anesthetize the rats on the 21 st day of the experiment, 8 h following the last dosage of APRE. The serum for biochemical analysis was separated by centrifuging blood samples taken from the abdominal aorta at 4000 *g* for 15 min (Salama et al. 2020). Quickly after blood was collected, the animals were sacrificed by cervical dislocation, and their kidneys were removed. The serum samples were kept at a temperature of − 80 °C until the biochemical tests were conducted. Each rat’s kidneys were fixed in 10% neutral-buffered formalin for histopathological and immunohistochemical studies. Further biochemical evaluation required the storage of the remaining kidney parts at − 80 °C. Renal tissues were homogenized using an ultrasonic homogenizer (SFX 550 Branson Digital Sonifier® ultrasonic cell disruptor/homogenizer, Danbury, CT, USA) for 2 min in ice-cold 10 mM potassium phosphate buffer (pH 7.4) at a 1:10 ratio (w/v), 100 mg per 1000 µl of buffer. The homogenates were then centrifuged at 10,000 g for 15 min at 4 °C, and the supernatants were collected for biochemical analysis (Mohyeldin et al. 2024).

### Biochemical assessment

#### Estimation of renal function

Serum urea and creatinine levels were measured using the following kits: 1,001,331, SPINREACT, Spain, and 11,734, Biosystems, Spain, respectively.

#### Evaluation of renal oxidative stress markers

Using a kit (MD 25 29, Bio Diagnostic, Egypt), we determined the MDA content in accordance with the manufacturer’s instructions. As directed by the manufacturer, GSH activity in tissue homogenate was measured using the GSH kit (GR 25 11, Bio Diagnostic, Egypt).

#### Estimation of renal inflammatory mediators

ELISA kits (Elabscience, USA) were used to detect renal TNF-α (E-EL-R2856) and IL-6 (E-EL-R0015) in accordance with the manufacturer’s instructions.

#### Assessment of renal Nrf2 and HO-1

ELISA kits (Elabscience, USA) were used to detect renal Nrf2 (E-EL-R1052) and HO-1 (E-EL-R0488) under the manufacturer’s instructions.

#### Assessment of renal cleaved caspase-3

Renal cleaved caspase-3 was detected using an ELISA kit (MBS1605634, MyBioSource, USA) following the manufacturer’s specifications.

### Quantitative real-time polymerase chain reaction (qRT-PCR)

Renal tissue was used to extract total RNA, and reverse transcription quantitative PCR (RT-qPCR) was carried out using previously established procedures (Mohyeldin et al. 2024; Sayed et al. 2025). Table 1 lists the primer sequences for the target genes. GAPDH was used as the internal reference gene, and the ^2−ΔΔ Ct^ technique was used to measure the levels of gene expression (Elmaidomy et al. 2023).

**Table 1:** The primer sequence

Genes	Sequence	
*Bax*	F	5′-CACGTCTGCGGGGAGTC-3′
	R	5′-TGTTGTCCAGTTCATCGCCA-3′
*Bcl-2*	F	5′-GGGCTACGAGTGGGATACTG-3′
	R	5′-GACCCCACCGAACTCAAAGA-3′
*GAPDH*	F	5′-CTCTCTGCTCCTCCCTGTTC-3′
	R	5′-CGACATACTCAGCACCAGCA-3′

### Immunodetection by Western blot

The tissue was washed with pre-cooled phosphate buffer solution to remove blood and debris, then homogenized with RIPA Lysis Buffer (containing PMSF and Na3VO4) at a ratio of 3:10 (tissue: buffer, 300 mg tissue per 1000 μL buffer) and lysed on ice for 30 min. Then, it was centrifuged at 12,000 rpm for 10 min at 4 °C, and the supernatant was collected to measure protein concentration through the BCA Method. According to the molecular weight of the target protein, the appropriate SDS-PAGE Gel was prepared by loading equal amounts of protein into the wells of the SDS-PAGE gel and separating by size. The proteins were transferred from the gel onto a PVDF membrane. The membrane was incubated with a blocking buffer to prevent nonspecific binding. The specific primary antibodies, including P38 (1:1000, ab170099, Abcam, UK), phosphorylated P38 (1:1000, phospho T180 + Y182, ab195049, Abcam, UK), TLR4 (1:1000, ab217274, Abcam, UK), Bax (1:1000, ab32503, Abcam, UK), and Bcl-2 (1:1000, ab194583, Abcam, UK) were added to bind the target protein and incubated overnight. The membrane was incubated for 2 h in a secondary antibody conjugated to a detection enzyme (HRP, 1:5000). Finally, the protein was visualized using the chemiluminescent ChemiDoc Imaging System from Bio-Rad, USA (Sharata et al. 2025a).

### Immunohistochemical staining

For immunohistochemical staining, 5-μm sections of renal tissue embedded in paraffin were deparaffinized and rehydrated. Antigen retrieval was performed by soaking the sections in a retrieval solution (Tris–EDTA, pH 9.0). Subsequently, they were placed in a water bath (98 °C) for 20 min to provide the unmasked antigens. A solution containing 3% hydrogen peroxide (H_2_O_2_) was applied for 15 min to inhibit endogenous peroxidase activity. The sections were exposed to 5% normal rabbit serum to prevent non-specific background staining. Prepared sections were exposed to primary antibodies, including NF-κB p65(1:200, bs-0465R, BiossAntibodies, China). The secondary antibody staining using a goat anti-rabbit biotinylated antibody for 20 min was applied to detect primary antibodies. Then, a 20-min incubation with prediluted (1:500) streptavidin horseradish peroxidase was done. Finally, DAB was used as a chromogen to observe antibody binding areas, and Mayer’s Hematoxylin was used to counterstain the samples. Visualization was performed using a light microscope to evaluate the degree of positive immunoreactivity in renal tissues (Abdelmawgood et al. 2025).

### Histological examination

Each animal’s kidney tissues were removed, and they were promptly preserved in 10% neutral-buffered formalin. The samples were then examined using a light microscope (Olympus, U.TV0.5XC-3) after being prepared and stained using conventional hematoxylin and eosin (H&E) procedures (Suvarna 2013). Group assignments were unknown at the time of all assessments and analyses. Histopathological alterations in renal tissue were categorized as 0: none, 1: mild, 2: moderate, and 3: severe, depending on the degree of inflammatory cell infiltration as well as renal tissue degradation and necrosis (Badreldin et al. 2024).

### Statistical analysis

The mean ± standard deviation (SD) of the data is displayed. The groups were statistically compared using one-way analysis of variance (ANOVA), and significant differences between the groups were then determined using Tukey’s post hoc test. GraphPad Prism (version 7; GraphPad Software Inc., USA) was used for all analyses. Moreover, for histopathological scoring data, which represent ordinal non-parametric data, the Kruskal–Wallis test was used to assess differences between groups, followed by Dunn’s multiple comparison test for pairwise comparisons. A statistically significant p-value is less than 0.05.

## Results

### Apremilast alleviated the altered renal function

**Fig. 2:**
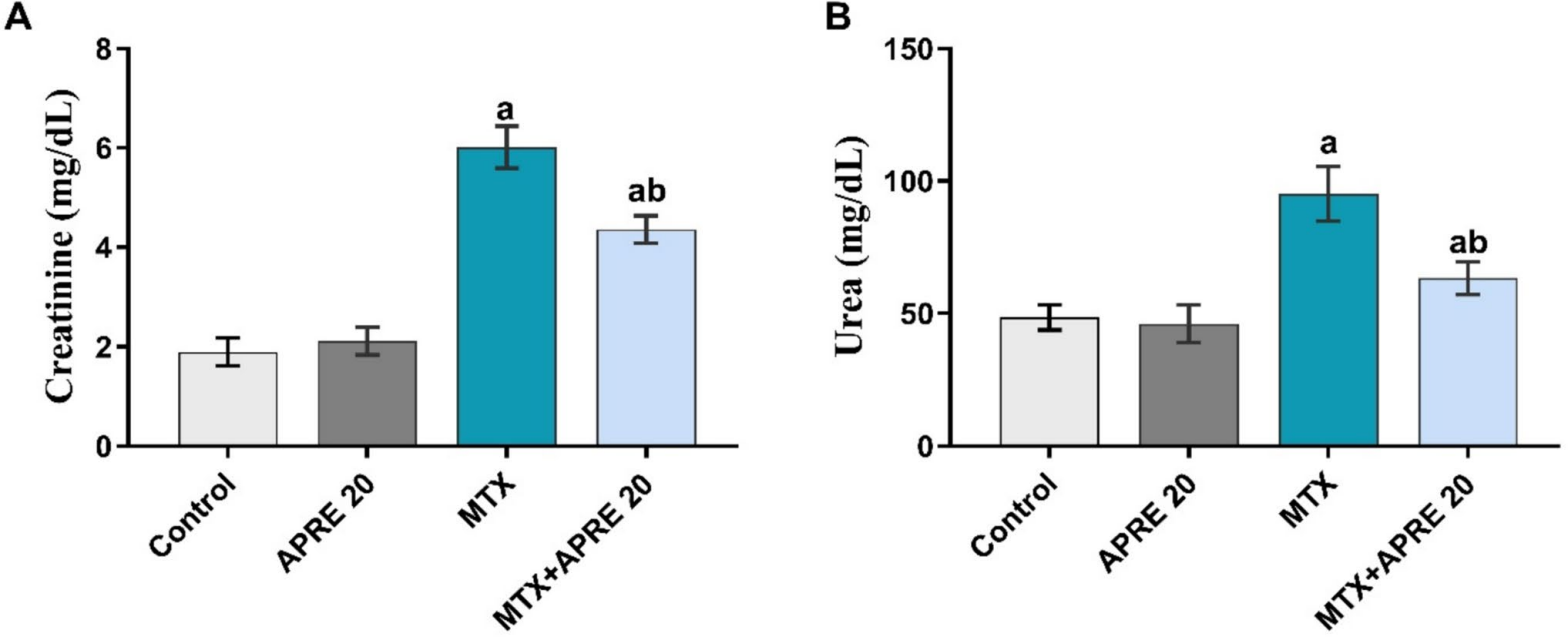
Apremilast’s impact on serum creatinine (**A**) and urea (**B**) levels. The mean ± standard deviation (*n* = 6). A significant difference was analyzed by one-way ANOVA, followed by the Tukey–Kramer post-analysis test. A statistically significant value is *P* < 0.05. two significant differences as compared to the other groups: (a) the control group and (b) the MTX group

As depicted in Fig. 2A, B, MTX significantly elevated the serum levels of creatinine and urea as compared to the control group. Conversely, the MTX + APRE group markedly attenuated these elevations compared to the MTX group.

### Apremilast attenuated the oxidative imbalance in renal tissue

**Fig. 3:**
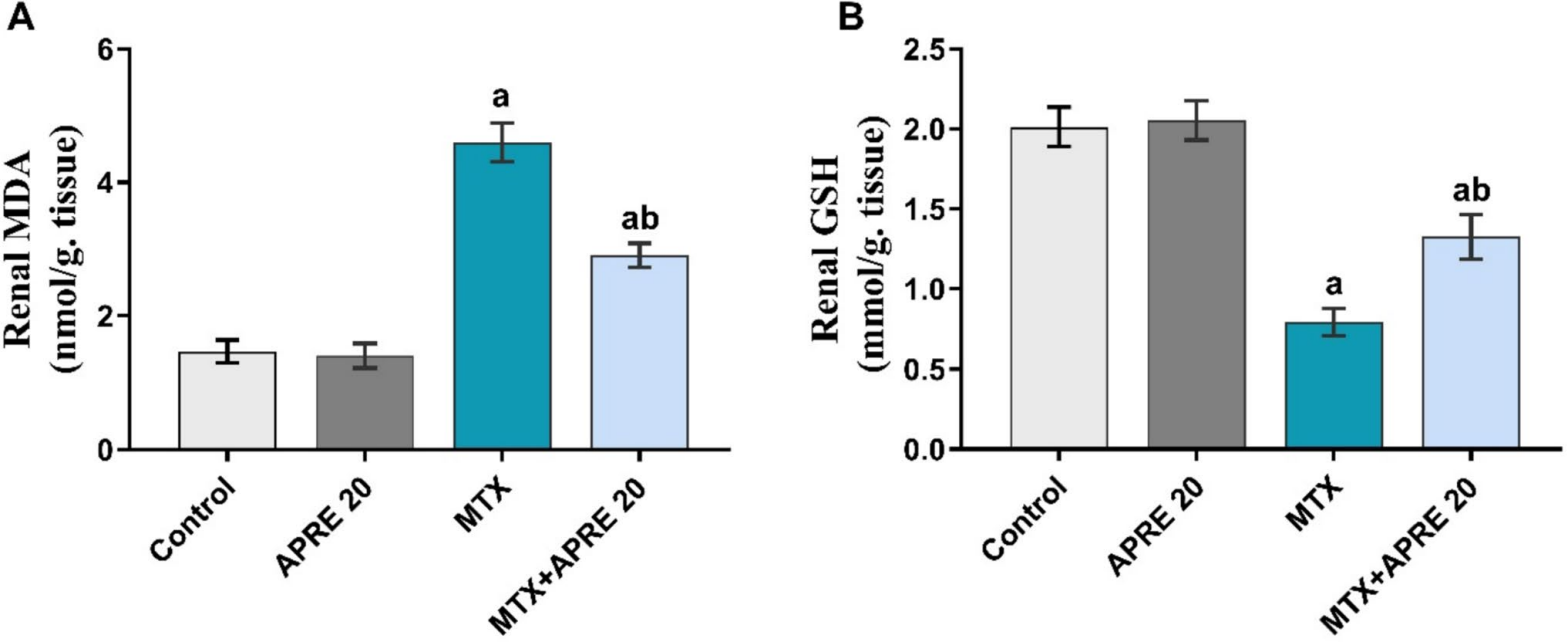
Apremilast’s impact on renal MDA (**A**) and GSH (**B**) levels. The mean ± standard deviation (*n* = 6). A significant difference was analyzed by one-way ANOVA, followed by the Tukey–Kramer post-analysis test. A statistically significant value is *P* < 0.05. two significant differences as compared to the other groups: (a) the control group and (b) the MTX group. GSH: reduced glutathione, MDA: malondialdehyde

To assess the renal antioxidant power of APRE, the renal levels of MDA and GSH were determined. The MTX group showed a significant increase in the renal MDA levels compared to the control group. However, rats treated with APRE exhibited a substantial inhibition in the renal contents of MDA, in contrast to the MTX group, as illustrated in Fig. 3A.

Furthermore, the renal GSH contents were significantly depleted after MTX administration compared to the control group. Contrariwise, the MTX + APRE group considerably increased the renal levels of GSH relative to the MTX group, as presented in Fig. 3B.

### Apremilast suppressed inflammatory markers (TNF-α and IL-6)

**Fig. 4:**
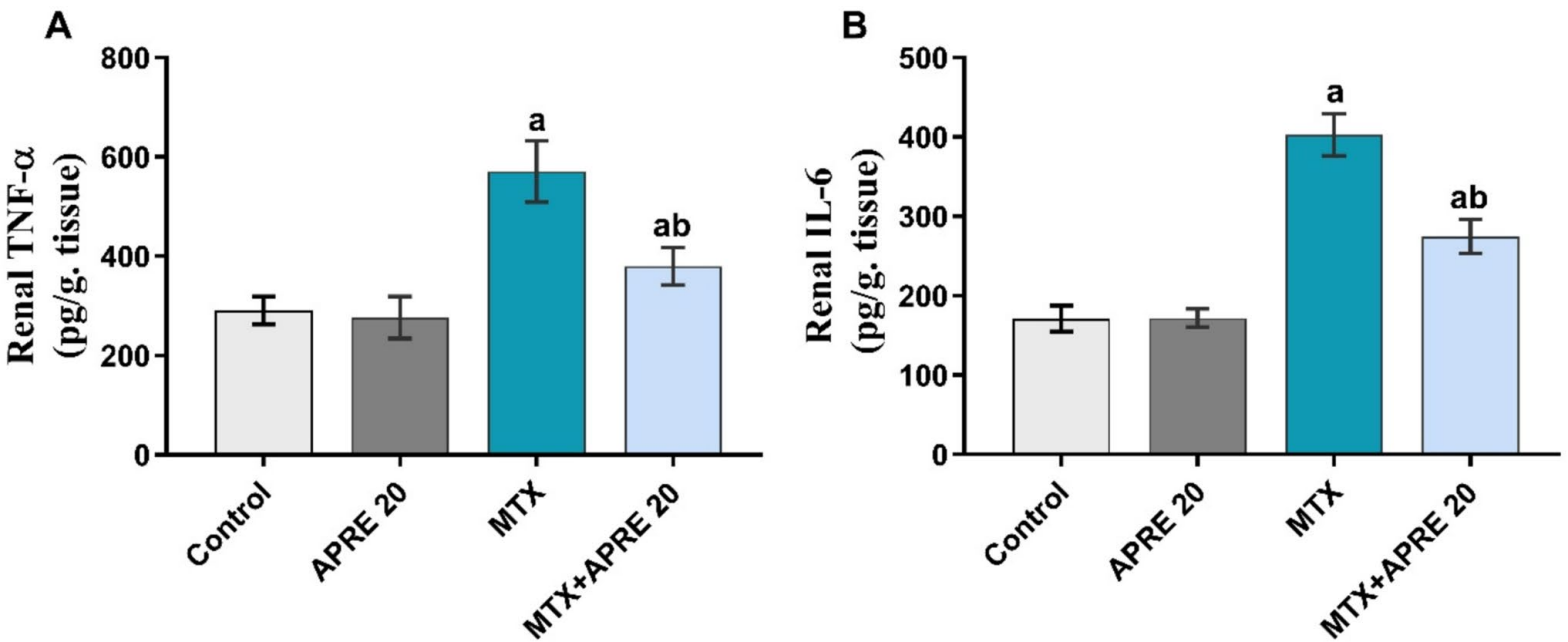
Apremilast’s impact on renal TNF-α (**A**) and IL-6 (**B**) levels. The mean ± standard deviation (*n* = 6). A significant difference was analyzed by one-way ANOVA, followed by the Tukey–Kramer post-analysis test. A statistically significant value is *P* < 0.05. two significant differences as compared to the other groups: (a) the control group and (b) the MTX group. IL-6: interleukin 6, TNF-α: tumor necrosis factor alpha

To confirm the anti-inflammatory impact of APRE, the renal pro-inflammatory levels (TNF-α and IL-6) were assessed. Figure 4A, B illustrates a significant upregulation in the renal levels of TNF-α and IL-6 in the MTX group compared to the control group. In contrast, APRE-treated rats showed a marked decline in the levels of these parameters compared to MTX-treated rats.

### Apremilast mitigated the apoptotic imbalance

**Fig. 5:**
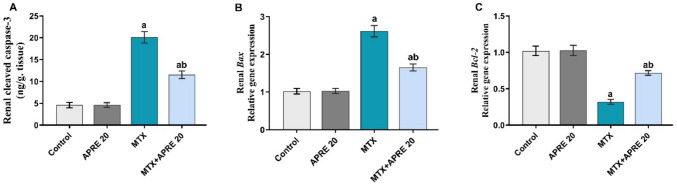
Apremilast’s impact on ELISA assay of renal cleaved caspase-3 (**A**), RT-PCR of Bax (**B**), and Bcl-2 (**C**) gene expression. The mean ± standard deviation (*n* = 6). A significant difference was analyzed by one-way ANOVA, followed by the Tukey–Kramer post-analysis test. A statistically significant value is *P* < 0.05. Two significant differences as compared to the other groups are: (a) the control group and (b) the MTX group. Bax: Bcl-2 associated X, Bcl-2: B-cell lymphoma 2

To determine the anti-apoptotic effect of APRE, the renal expression of *Bax* and *Bcl-2* genes was evaluated, as well as the renal expression of the cleaved caspase-3 protein was detected. As depicted in Fig. 5A–C, the renal mRNA level of *Bax* and the renal caspase-3 protein level showed a significant increase, while a marked decrease in the renal mRNA levels of *Bcl-2* was observed in MTX rats compared to the control rats. Conversely, the APRE administration substantially decreased the renal mRNA *Bax* level and caspase-3 protein level, whereas it significantly increased the renal mRNA *Bcl-2* level compared to the MTX group.

### Apremilast modulated Nrf2/HO-1 signaling cascade

**Fig. 6:**
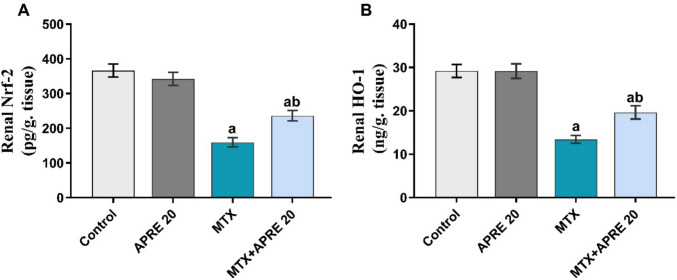
Apremilast’s impact on renal Nrf-2 (**A**) and HO-1 (**B**) levels. The mean ± standard deviation (*n* = 6). A significant difference was analyzed by one-way ANOVA, followed by the Tukey–Kramer post-analysis test. A statistically significant value is *P* < 0.05. two significant differences as compared to the other groups: (a) the control group and (b) the MTX group. HO-1: heme oxygenase 1, Nrf-2:nuclear Factor Erythroid 2-related Factor 2

The renal levels of Nrf-2 and HO-1 showed a marked reduction after MTX administration compared to the control group. Whereas the APRE-treated rats exhibited a considerable rise in their levels compared to the MTX group, as illustrated in Fig. 6A, B.

### Apremilast inhibited TLR4/P38 MAPK/NF-κB p65/Bax/Bcl-2 signaling pathway

**Fig. 7:**
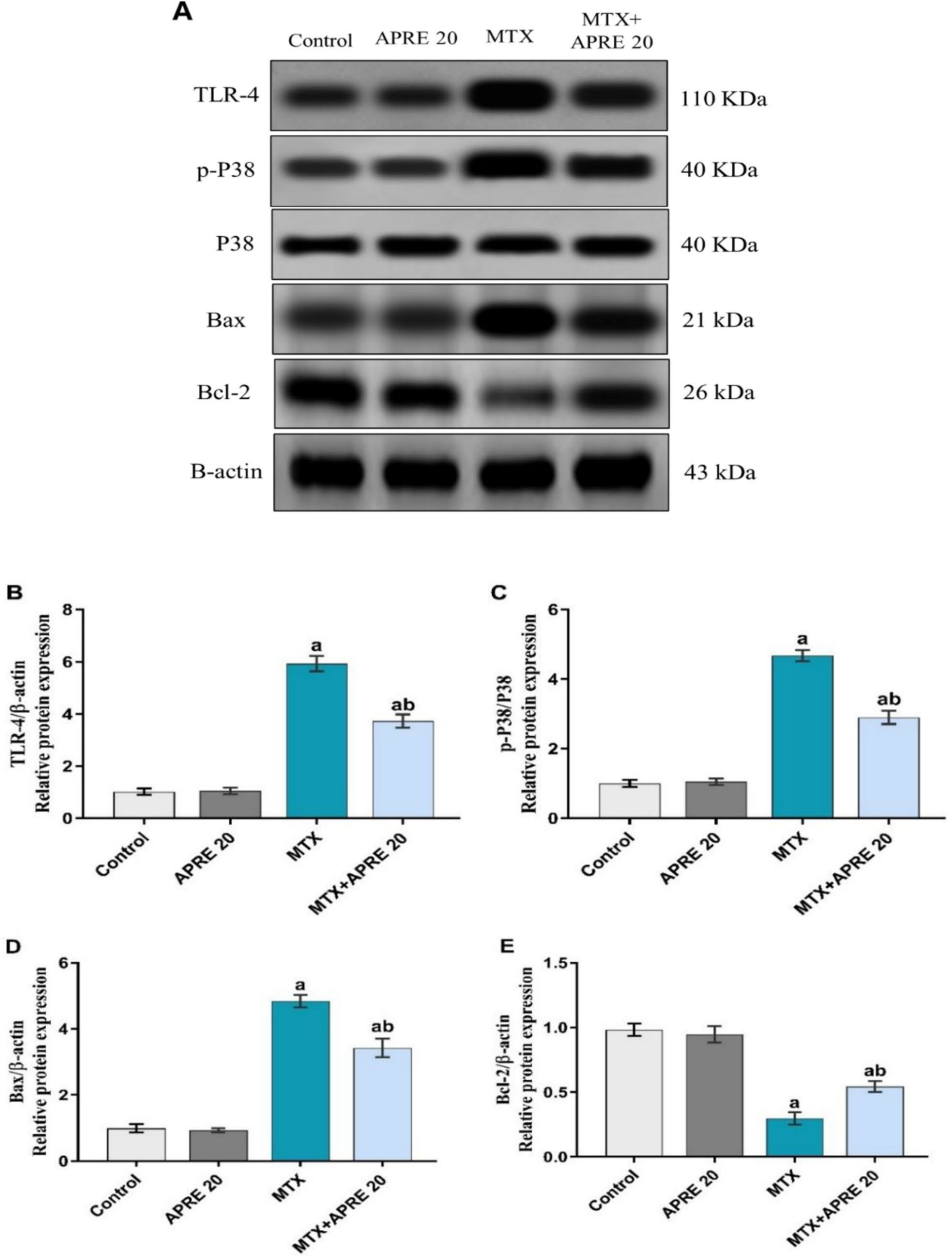
Immunoblotting graphs showing apremilast’s impact on the expression of renal TLR4, p-P38 MAPK, Bax, and Bcl-2 (**A**), protein expression levels of renal TLR4 (**B**), p-P38 MAPK (**C**), Bax (**D**), and Bcl-2 (**E**). The mean ± standard deviation (*n* = 3). A significant difference was analyzed by one-way ANOVA, followed by the Tukey–Kramer post-analysis test. A statistically significant value is *P* < 0.05. two significant differences as compared to the other groups: (a) the control group and (b) the MTX group. Bax: Bcl-2 associated X, Bcl-2: B-cell lymphoma 2, TLR-4: toll like receptor 4

**Fig. 8:**
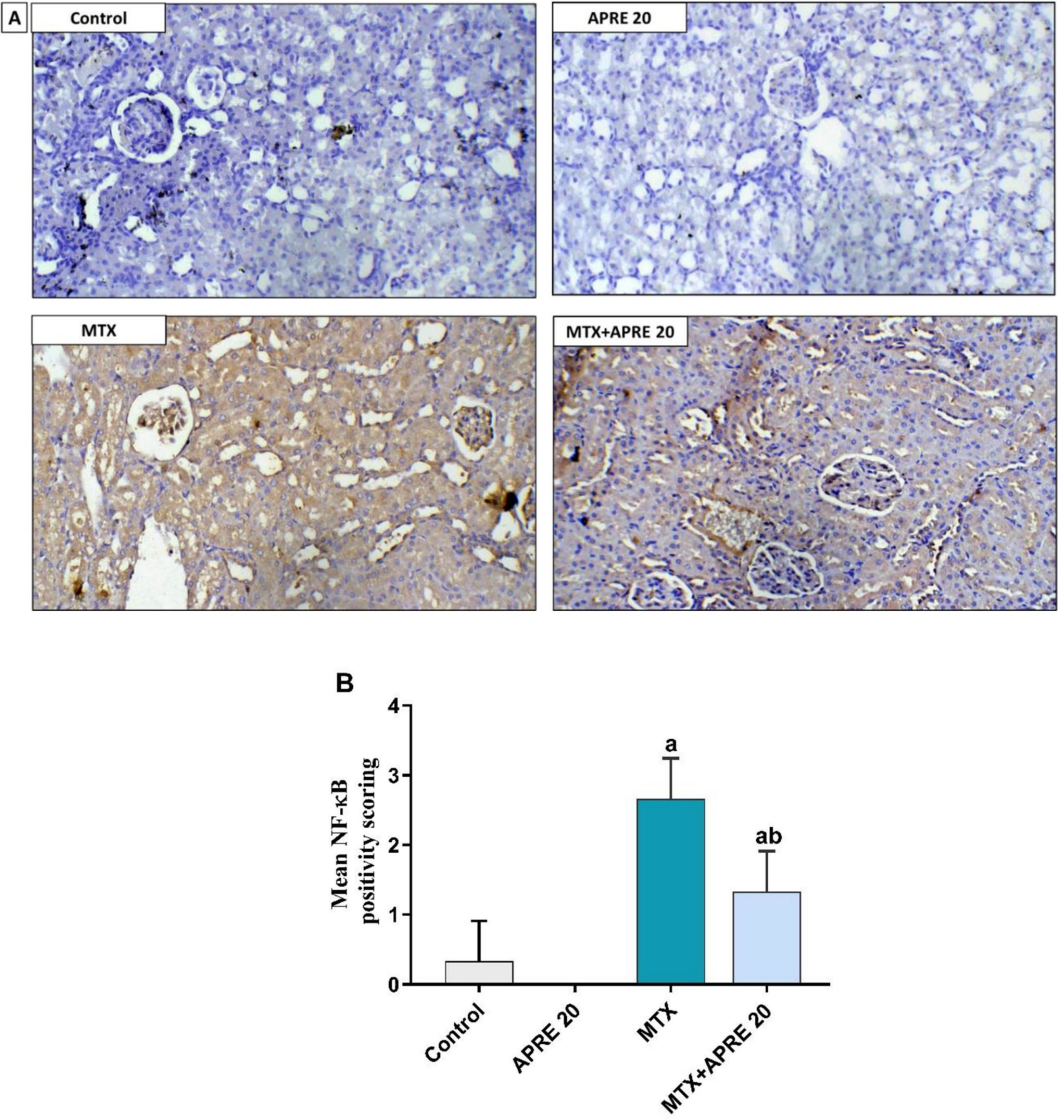
Photomicrographs showing the impact of apremilast on the expression of renal NF-κB p65 (× 200) (**A**), NF-κB p65 scoring (**B**). The mean ± standard deviation (*n* = 3). A significant difference was analyzed by one-way ANOVA, followed by the Tukey–Kramer post-analysis test. A statistically significant value is *P* < 0.05. Two significant differences as compared to the other groups: (a) the control group and (b) the MTX group. NF-κB: nuclear factor kappa B

The western method was used to measure the protein expression of renal TLR4, p-P38, Bax, and Bcl-2, as shown in Fig. 7A–E, whereas immunohistochemistry was used to determine NF-κB p65 expression, as shown in Fig. 8.

MTX group exhibited a significant rise in the protein level of TLR-4, Bax, NF-κB p65, as well as a marked activation of p-P38 protein, whereas a significant downregulation of the Bcl-2 protein was observed compared to the control group. Conversely, the APRE administration attenuated these alterations compared to the MTX group.

### Apremilast prevented MTX-induced renal tissue injury

**Fig. 9:**
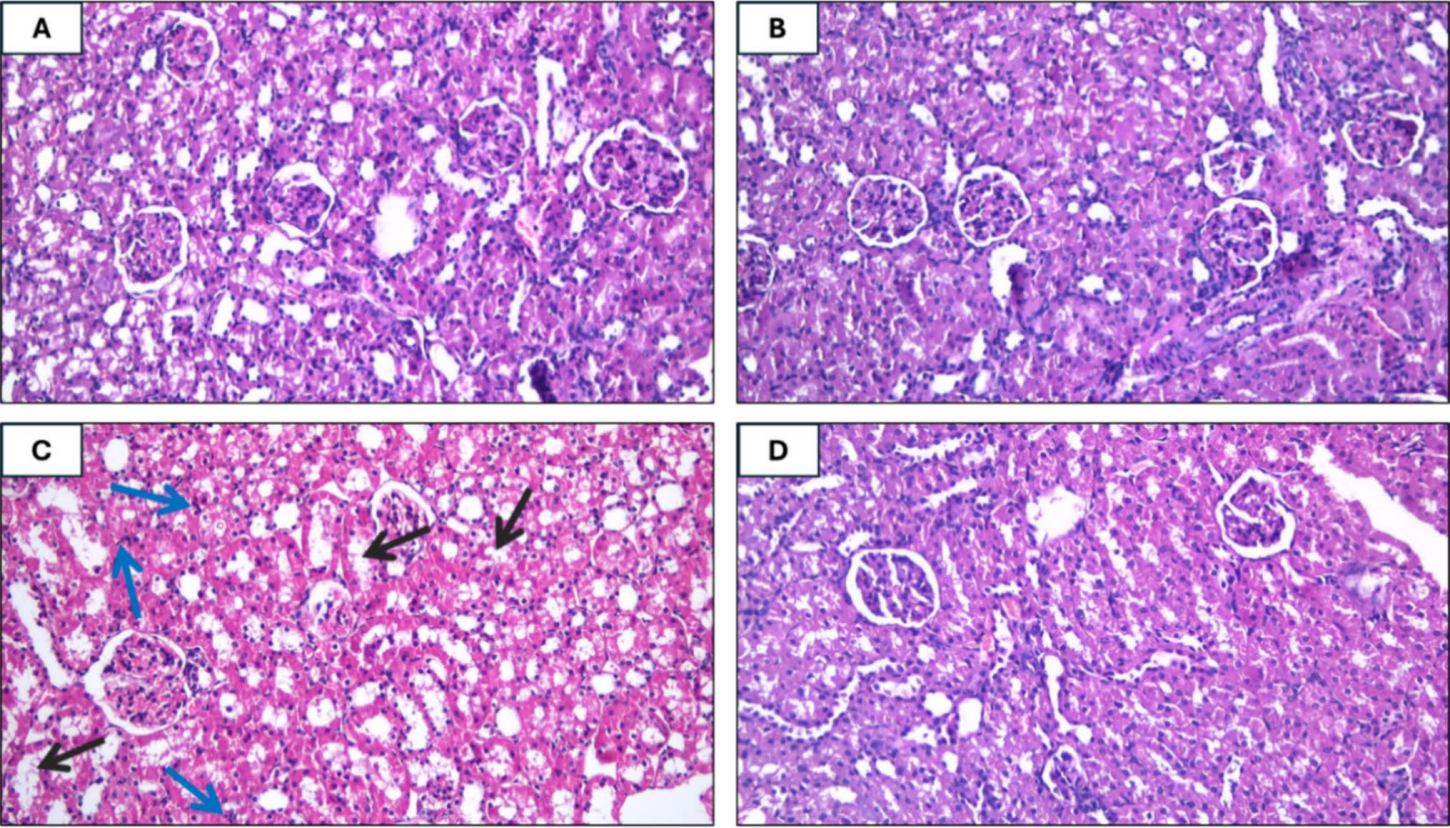
Representative photomicrographs of renal tissues (H&E staining, × 200) of the control (**A**), APRE (**B**), MTX (**C**), MTX + APRE (**D**) groups. blue arrow: inflammation, black arrow: vacuolar degeneration and necrotic tubular epithelial cells

The renal tissues of the MTX group exhibited a significant increase in inflammation, vacuolar degeneration, and necrotic tubular epithelial cells in comparison to control and APRE groups, which exhibited normal renal tissues, as illustrated in Fig. 9A–D and Table 2. In contrast, treatment with APRE resulted in a substantial improvement of these changes as compared to the MTX group.

**Table 2:** Histological scoring for the different groups

Groups	Control	APRE	MTX	MTX + APRE
Inflammation	0	0	1	0
Vacuolar degeneration	0	0	2	1
Tubular necrosis	0	0	2	1
Total scoring	0	0	5^a^	2^b^

The significance of differences was assessed using the Kruskal–Wallis test, followed by Dunn’s multiple comparison test (*n* = 6), where ^a^ indicates a significant difference relative to the control group and ^b^ indicates a significant difference relative to the MTX group

## Discussion

MTX is a frequently prescribed medication for the treatment of malignancies such as acute lymphoblastic leukemia, non-Hodgkin lymphoma, osteosarcoma, and breast cancer (Sakura et al. 2018), and numerous autoimmune diseases, including rheumatoid arthritis, psoriasis, and psoriatic arthritis (Khan et al. 2012; Kim et al. 2017). MTX cytotoxicity is not limited to tumor cells; it may also impact healthy cells in crucial organs. The kidney is the primary organ impacted, exhibiting hallmarks of renal toxicity (Tousson et al. 2014; Hadi et al. 2012). The mechanisms behind MTX-induced renal damage have been elucidated via diverse pathways, including inflammation, oxidative stress, and apoptotic pathways (Roghani et al. 2020; Ulusoy et al. 2016). We envisioned that APRE, often used with MTX in certain situations for psoriasis therapy, may provide protection against MTX-induced kidney injury. Indeed, we exhibited significant renal injury induced by MTX (as evidenced by elevated serum creatinine and urea levels and histopathological alterations) and its amelioration with the co-administration of APRE. The investigation also highlights oxidative stress, inflammation, and apoptosis as targets for mitigating MTX-induced tissue damage.

The present investigation demonstrates that MTX induces abnormalities in renal functions, as shown by substantial elevations in biomarkers such as creatinine and urea. The increased concentrations of biochemical markers are often associated with the release of enzymes into the bloodstream due to alterations in cell membrane permeability and the degradation of structural integrity (Sherif et al. 2019). This was corroborated by the histological alterations in the kidney tissues, which were consistent with numerous prior studies that demonstrated the deleterious effects of MTX on both organs (Dar et al. 2021; Pınar et al. 2018; Asci et al. 2017). A concerning finding is that pretreatment with APRE led to a substantial decrease in creatinine and urea, simultaneously ameliorating all renal histological anomalies induced by MTX.

Raised levels of reactive oxygen species (ROS) are associated with MTX-induced oxidative stress (Eki̇nci̇-Akdemi̇r et al. 2018; Sharata 2025). In the current study, the oxidative damage of MTX was evidenced by increased lipid peroxidation expressed by elevated MDA as a secondary product and decreased antioxidant activity due to the decreased GSH. Specifically, decreased GSH expression may impair the antioxidant defense mechanism (Khafaga and El-Sayed 2018; Ibrahim et al. 2025). Previous investigations corroborated our results, demonstrating that rats treated with MTX had elevated renal MDA levels and decreased GSH levels (Arab et al. 2022; Zahran et al. 2025). Surprisingly, APRE dramatically decreased the oxidative stress brought on by MTX, indicating its protective impact against the production of free radicals, which is consistent with earlier research that documented APRE’s antioxidant qualities (Al-Harbi et al. 2023; Al-Harbi et al. 2022).

The present investigation demonstrated that MTX resulted in a substantial elevation of TNF-α and IL-6 levels in renal tissues. These interleukins induce inflammation and tissue necrosis by triggering the apoptotic cascade (Tacke et al. 2009; Abdel-Daim et al. 2017). The increased levels of pro-inflammatory cytokines correlated with the upregulation of renal NF-κB and caspase-3 expression, hence substantiating organ damage. These results align with previous investigations that indicated elevated levels of TNF-α and IL-6 after MTX intoxication (Kalantari et al. 2024; Abraham et al. 2010). The beneficial action of APRE against MTX-induced kidney injury is demonstrated by the downregulation of inflammatory cytokines and the tissue expression of NF-κB and caspase-3. The results obtained can be explained based on the prior investigations that documented the anti-inflammatory effects of APRE in several experimental studies (Imam et al. 2018a; Yin et al. 2021).

Additionally, several previous studies have proven the role of apoptosis in MTX-evoked renal injury (Dar et al. 2021; Soliman et al. 2020), so we evaluated the effect of APRE on the Bax/Bcl-2/caspase-3 pathway. The Bcl-2 family comprises Bax and Bcl-2, which affect cellular vulnerability to apoptosis (Almeida et al. 2000; Abdelnaser et al. 2023). Bcl-2 represses apoptosis (Yang et al. 1997; Abdelnaser et al. 2025b). In contrast, Bax induces apoptosis when it is subjected to oxidative stress and inflammation (Mahmoud et al. 2019; Abdelnaser et al. 2024). MTX administration increased the levels of Bax and caspase-3 in the kidneys, while decreasing the expression of Bcl-2. Fortunately, pre-administration of APRE restored the above changes, thereby alleviating MTX-induced apoptosis. These results are consistent with prior research indicating the inhibitory effect of APRE on apoptosis (Yin et al. 2021; Imam et al. 2016).

For further elucidation of the suggested protective mechanism of APRE against nephrotoxicity evoked by MTX, an assessment of the expression of the renal Nrf2/HO-1 signaling pathway was carried out. Nrf2 regulates the antioxidant response, which encompasses numerous downstream genes that are responsible for regulating oxidative stress (Li et al. 2017), which regulates HO-1 and cellular defensive mechanisms (Ge et al. 2017). Our results demonstrated that the expression of Nrf2 and HO-1 is substantially reduced in response to MTX administration, suggesting that these proteins play a critical role in the regulation of oxidative stress in the kidneys. Consistent with our findings, recently published evidence corroborated the inhibitory effects of MTX on Nrf2/HO-1 signaling (Mahmoud et al. 2017; Radwan et al. 2023). Surprisingly, APRE caused a marked elevation in the expression of Nrf2 and HO-1, which runs concurrently with the article of Al-Harbi, N.O., et al., which reported that the Nrf2/HO-1 signaling pathway was regarded as a potential mechanism for elucidating the efficacy of APRE in reducing acute lung injury induced by LPS (Al-Harbi et al. 2023).

The present study suggested the possible contribution of TLR4 in MTX-induced nephrotoxicity cascades, so we assessed the impact of APRE on the TLR4/NF-κB p65/P38-MAPK cascade. TLR4 is the best-recognized pattern recognition receptor that triggers an inflammatory response as a component of innate immune defense, activated by endogenous damage-associated molecular patterns (DAMPs) from necrotic cells (Sharata et al. 2025a; Sharata et al. 2025b; Kiziltas 2016). Conversely, the activation of TLR4 has been observed to promote the production of ROS through NADPH oxidase, suggesting a fascinating crosstalk between ROS and TLR4 (Park et al. 2004). When TLR4 is activated, NF-κB p65 is phosphorylated and translocated, which encourages the synthesis of inflammatory mediators, including IL-6 and TNF-α (Rofaeil et al. 2025; Molteni et al. 2016). Furthermore, TLR4 may activate MAP kinases, particularly P38 (Sharata et al. 2025a). This further influences Bcl-2 activity, particularly under stress conditions, eventually resulting in enhanced apoptosis (El-Sheikh et al. 2015; Cheng et al. 2016). MTX administration resulted in dramatic upregulation of the TLR4/NF-κB p65/P38-MAPK cascade, and these findings concur with previous studies (Hassanein et al. 2023). Contrariwise, pretreatment with APRE caused a significant downregulation of the TLR4/NF-κB p65/P38-MAPK cascade, and these results obtained by APRE align with previous studies that demonstrated the cardioprotective effect of APRE against cardiotoxicity caused by doxorubicin via modulating the NF-κB p65/P38-MAPK cascade (Imam et al. 2016; Imam et al. 2018b).

The current research presents APRE as a potentially effective treatment strategy to lessen MTX-induced kidney damage, particularly in psoriasis patients. Additionally, to fully assess the therapeutic efficacy of APRE in reducing MTX-induced renal injury, these unique results must be validated in future studies employing siRNA-mediated gene silencing or specific pathway inhibitors and activators, which would provide definitive causal evidence for these pathway roles. Clinical trials using a range of APRE administration dosages and durations would also be warranted.

## Conclusion

**Fig. 10:**
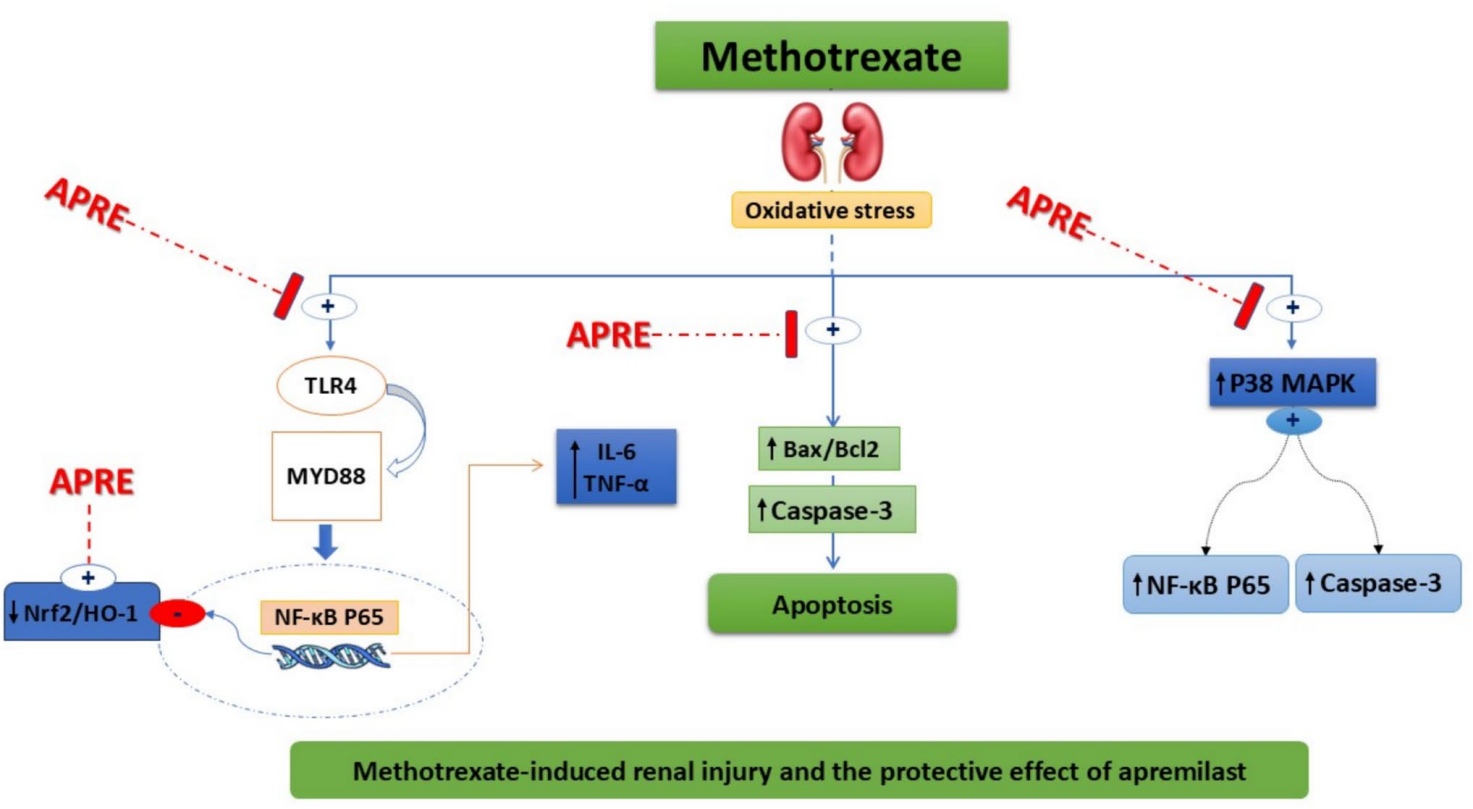
The mechanistic approach by which Apremilast attenuated MTX-induced nephrotoxicity

The present work demonstrated the antioxidant, anti-inflammatory, and anti-apoptotic properties of APRE in mitigating MTX-induced nephrotoxicity in rats, potentially via many complementary pathways. Firstly, through the direct suppression of the TLR4/NF-κB p65/P38-MAPK signaling pathway. Secondly, via stimulation of Nrf2/HO-1 signaling, which in turn repressed oxidative damage and apoptosis, as depicted in Fig. 10.

## Supplementary Information

Below is the link to the electronic supplementary material.

ESM 1 (PDF 226 KB)

ESM 2 (PDF 274 KB)

## Data Availability

All data produced or examined during this investigation are incorporated in this published article.
