# Supplementary material for: Apremilast ameliorates methotrexate-induced renal injury in rats: role of TLR4/NF-κB/P38 MAPK/caspase-3 and Nrf2/HO-1 signaling pathways
Source: Naunyn Schmiedebergs Arch Pharmacol. 2025 Dec 15;399(5):7615–29. doi: 10.1007/s00210-025-04846-w (PMC13053451; doi:10.1007/s00210-025-04846-w)
Supplement: Supplementary file 2 — (PDF 274 KB) [file 210_2025_4846_MOESM2_ESM.pdf]

Renal p-P38

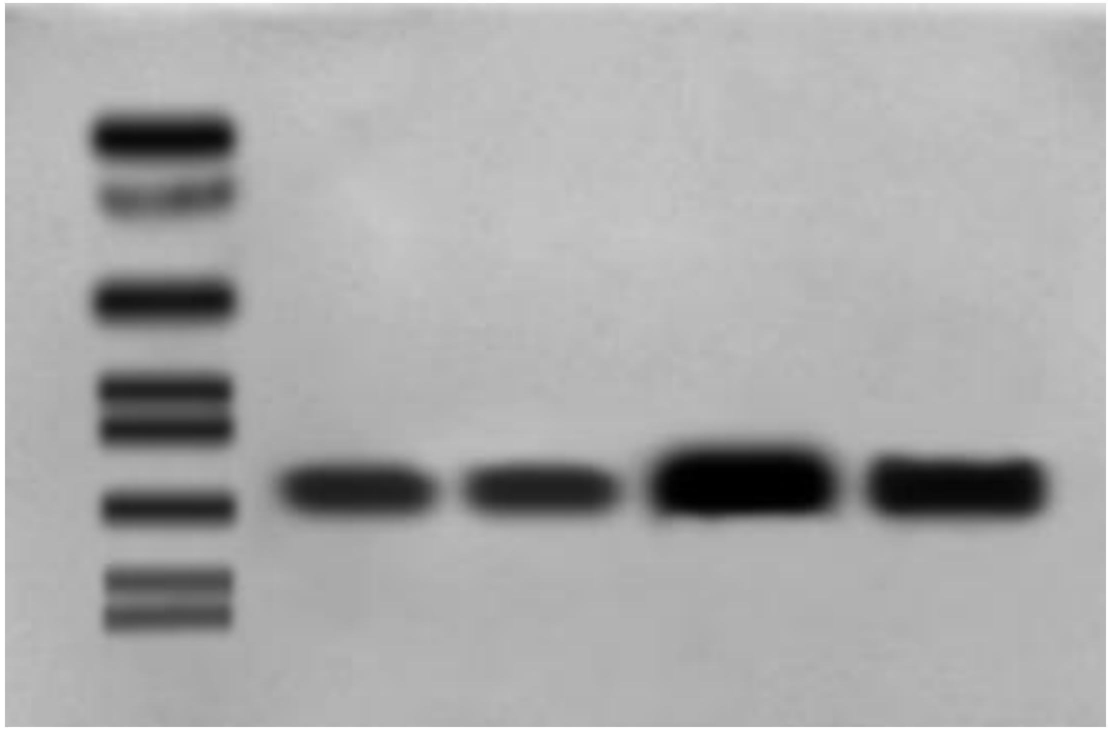

p-P38 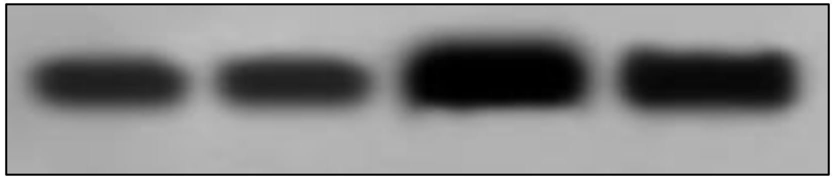 40 KDa

Renal P38

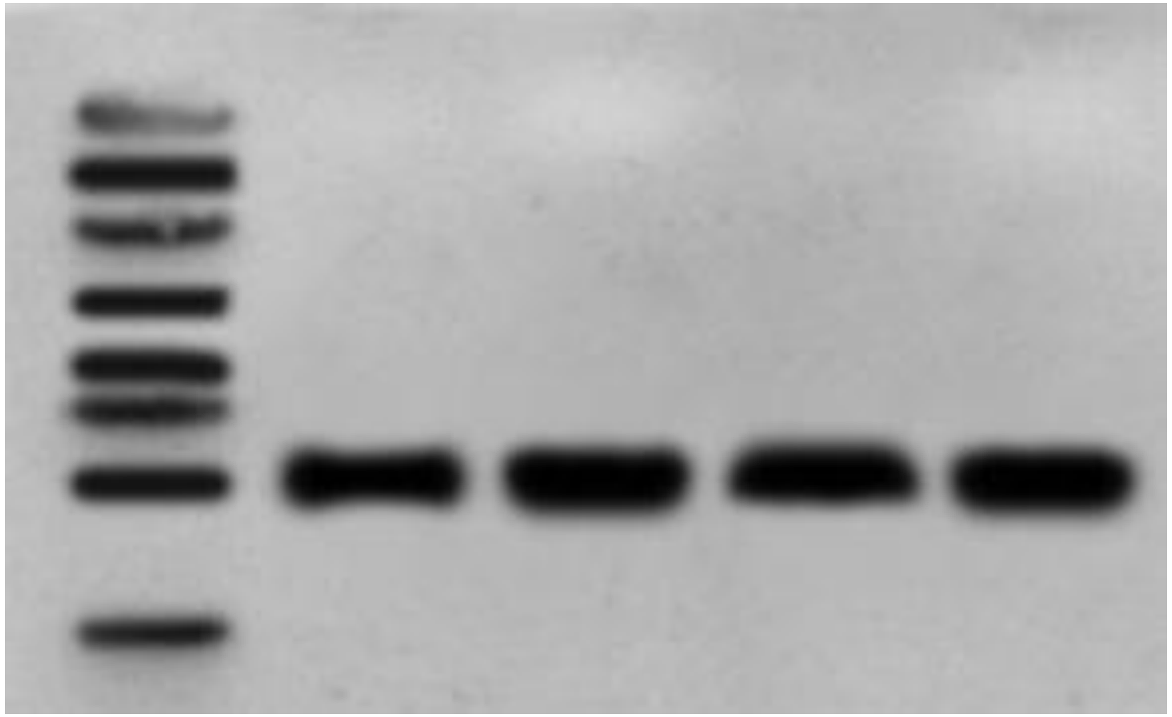

P38 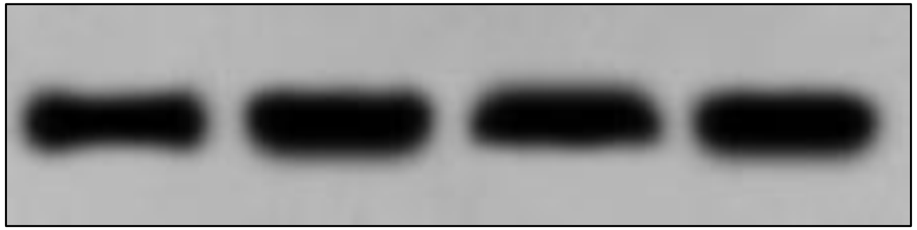 40 KDa

Renal TLR-4

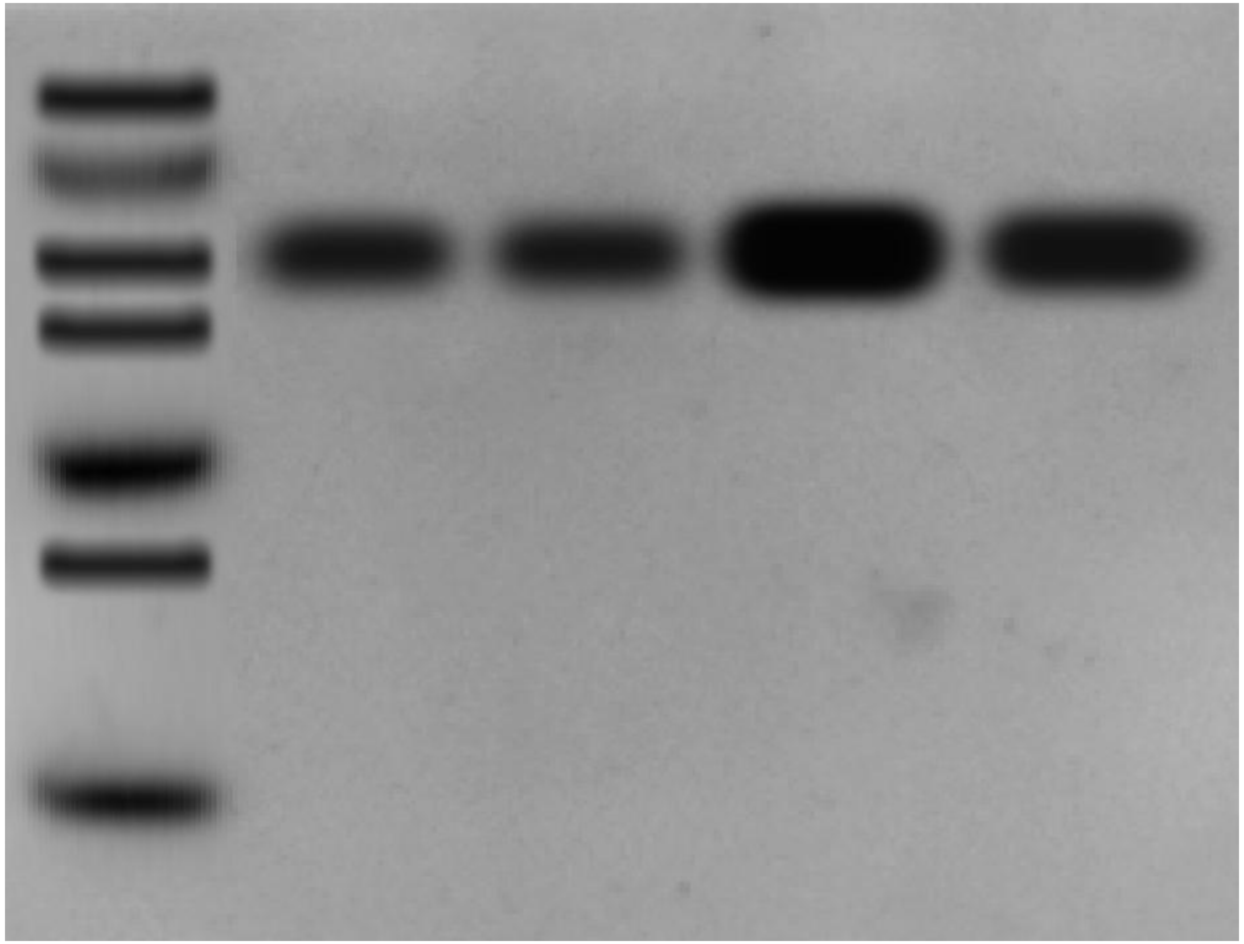

TLR-4

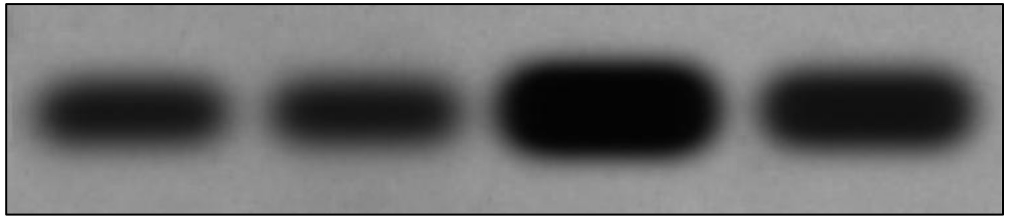

110 KDa

Renal B-actin

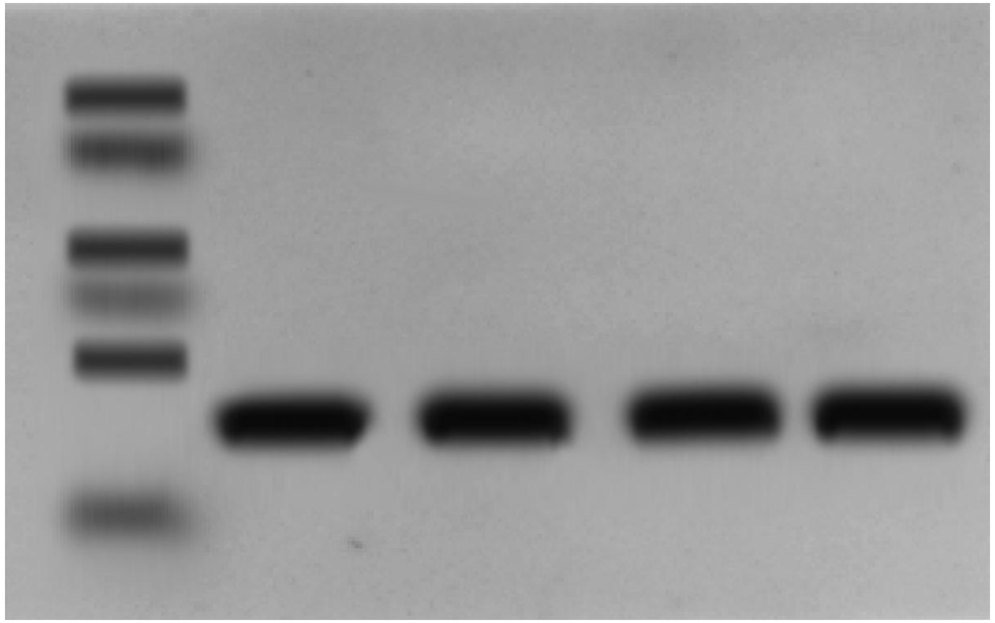

B-actin 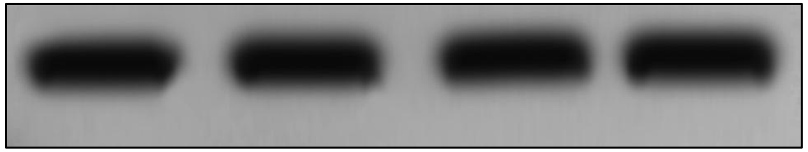 43 kDa

Renal Bax

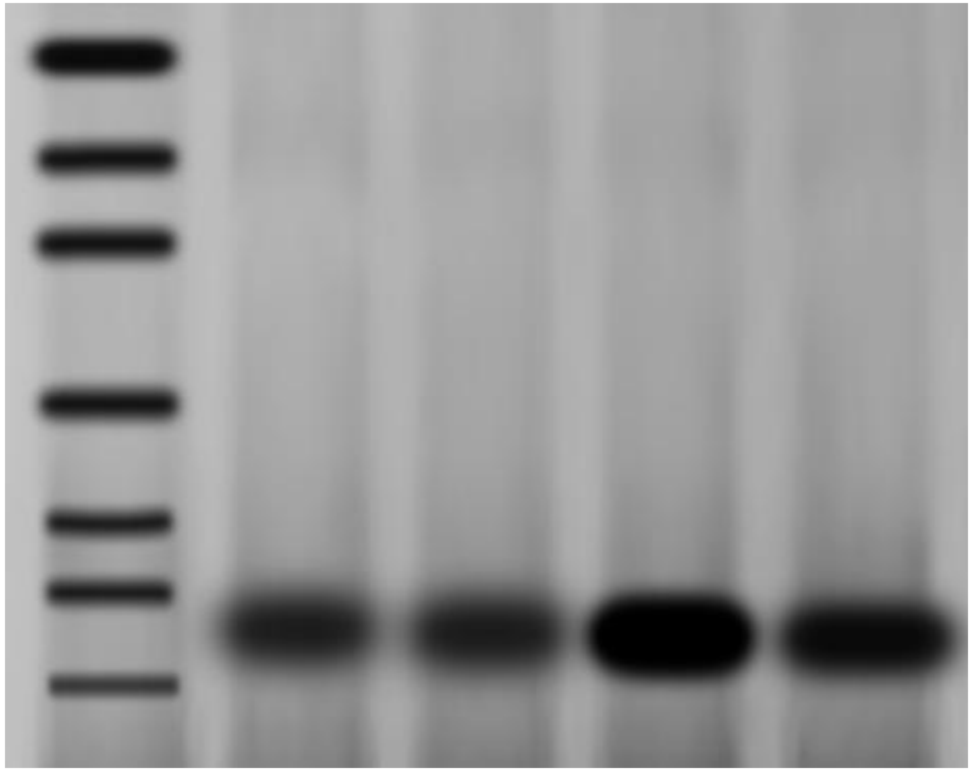

Bax

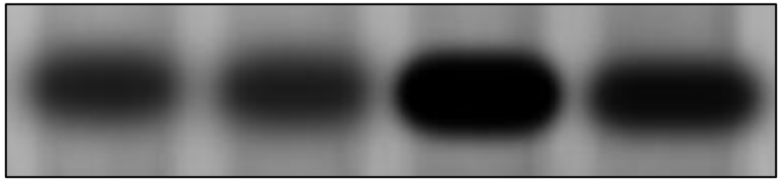

21 kDa

<u>Renal Bcl-2</u>

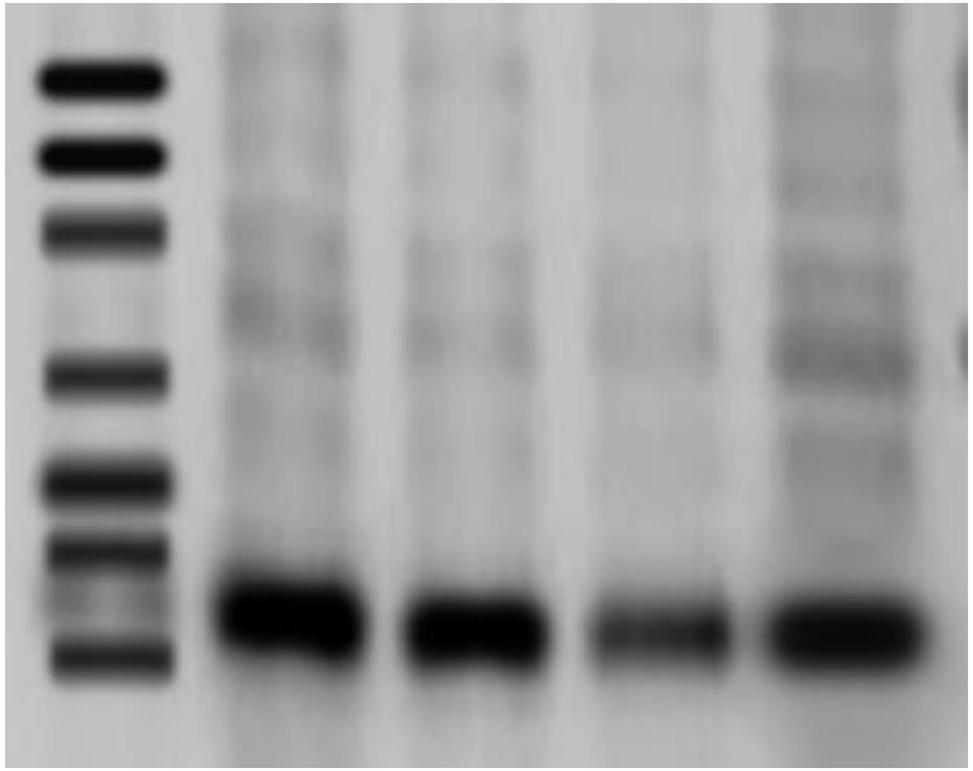

Bcl-2

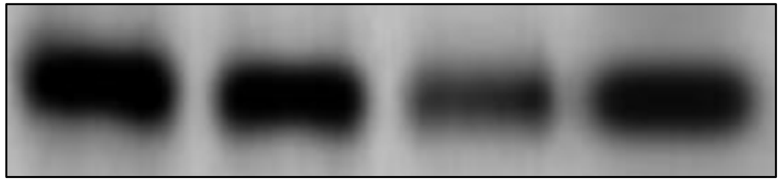

26 kDa

Renal B-actin

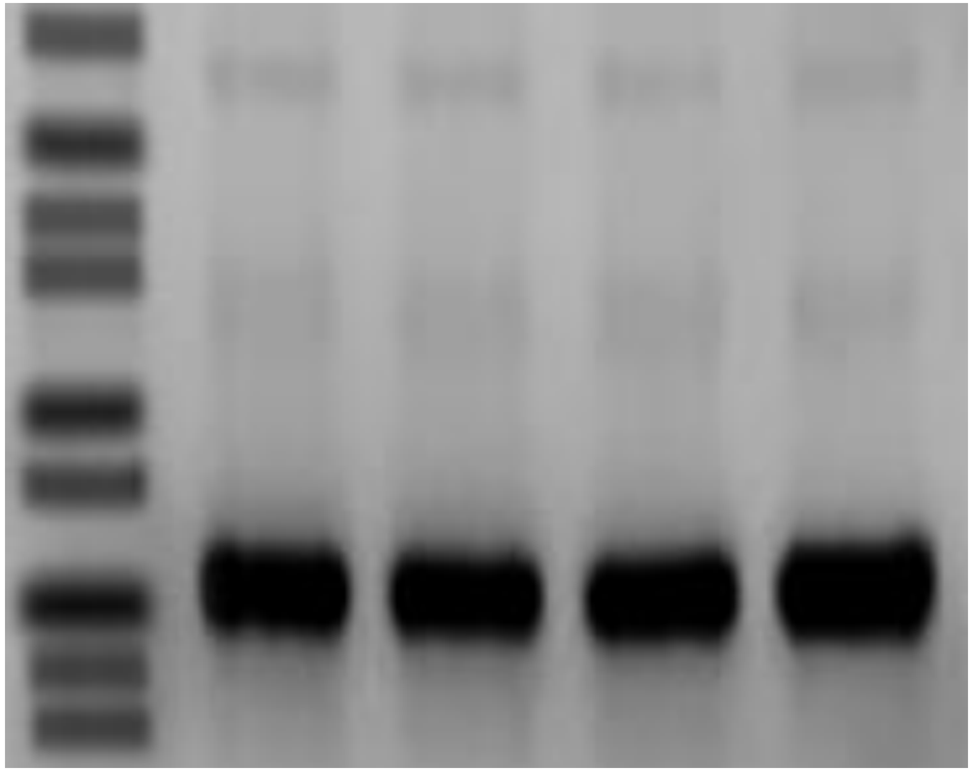

B-actin

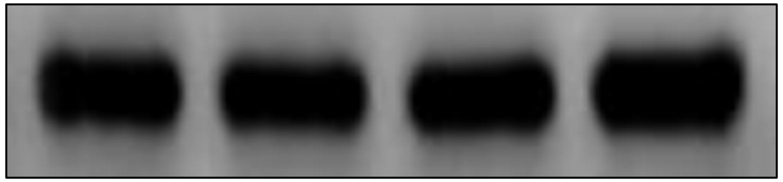

43 kDa

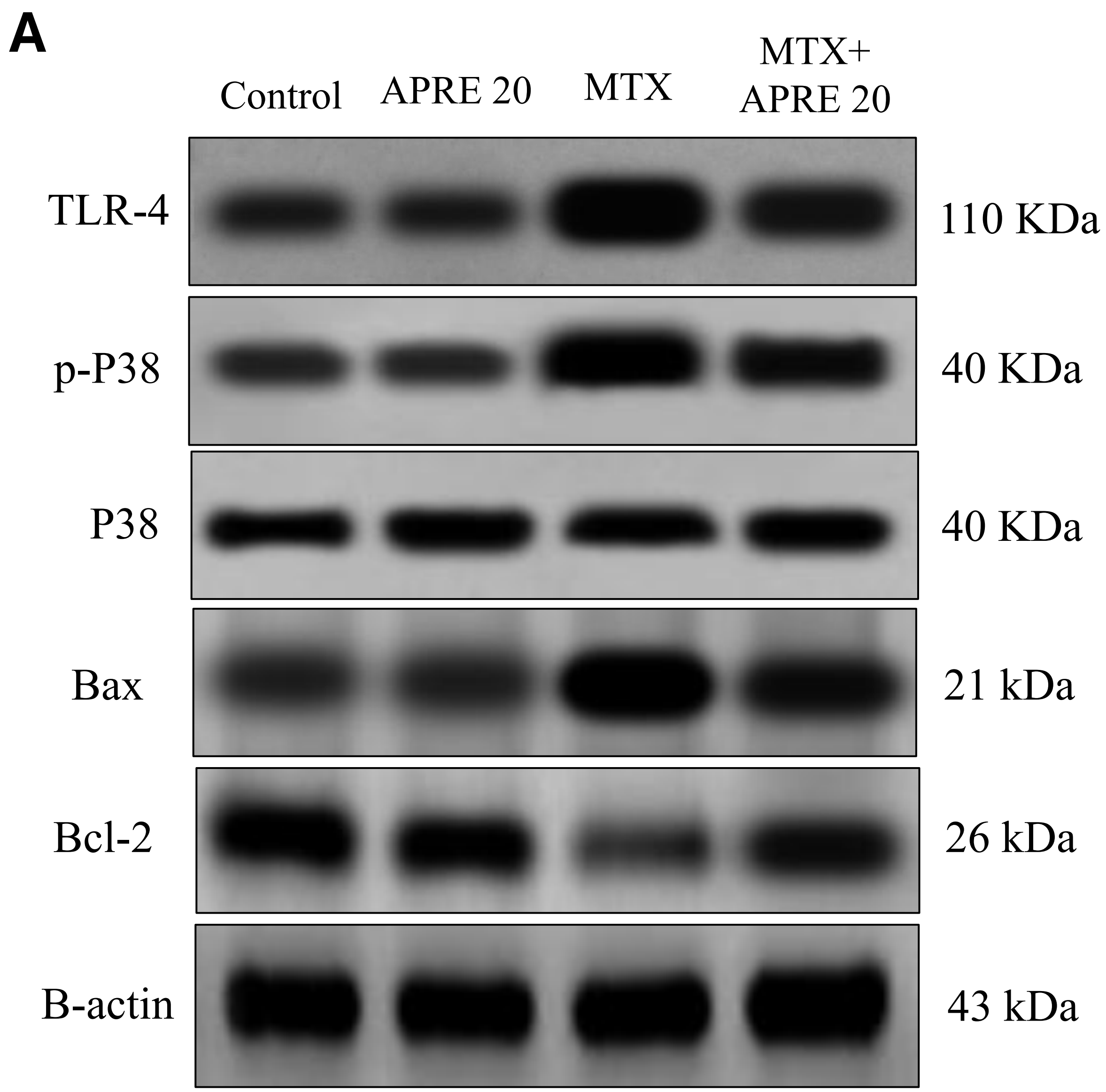
A
Control
APRE 20
MTX
MTX+
APRE 20
TLR-4
p-P38
P38
Bax
Bcl-2
B-actin
110 KDa
40 KDa
40 KDa
21 kDa
26 kDa
43 kDa
